# Calcium-Dependent Src Phosphorylation and Reactive Oxygen Species Generation Are Implicated in the Activation of Human Platelet Induced by Thromboxane A2 Analogs

**DOI:** 10.3389/fphar.2018.01081

**Published:** 2018-09-26

**Authors:** Pietro Minuz, Alessandra Meneguzzi, Laura Fumagalli, Maurizio Degan, Stefano Calabria, Roberta Ferraro, Marco Ricci, Dino Veneri, Giorgio Berton

**Affiliations:** ^1^Section of Internal Medicine, Department of Medicine, University of Verona, Verona, Italy; ^2^Section of General Pathology, Department of Medicine, University of Verona, Verona, Italy; ^3^Section of Haematology, Department of Medicine, University of Verona, Verona, Italy

**Keywords:** thromboxane A2, platelets, tyrosine kinase, calcium, NADPH oxidase

## Abstract

The thromboxane (TX) A_2_ elicits TP-dependent different platelet responses. Low amounts activate Src kinases and the Rho–Rho kinase pathway independently of integrin α_IIb_β_3_ and ADP secretion and synergize with epinephrine to induce aggregation. Aim of the present study was to investigate the role Src kinases and the interplay with calcium signals in reactive oxygen species (ROS) generation in the activatory pathways engaged by TXA_2_ in human platelets. All the experiments were performed *in vitro* or *ex vivo*. Washed platelets were stimulated with 50–1000 nM U46619 and/or 10 μM epinephrine in the presence of acetylsalicylic acid and the ADP scavenger apyrase. The effects of the ROS scavenger EUK-134, NADPH oxidase (NOX) inhibitor apocynin, Src kinase inhibitor PP2 and calcium chelator BAPTA were tested. Intracellular calcium and ROS generation were measured. Platelet rich plasma from patients treated with dasatinib was used to confirm the data obtained *in vitro*. We observed that 50 nM U46619 plus epinephrine increase intracellular calcium similarly to 1000 nM U46619. ROS generation was blunted by the NOX inhibitor apocynin. BAPTA inhibited ROS generation in resting and activated platelets. Phosphorylation of Src and MLC proteins were not significantly affected by antioxidants agents. BAPTA and antioxidants reduced P-Selectin expression, activation of integrin α_IIb_β_3_and platelet aggregation. TXA_2_-induced increase in intracellular calcium is required for Src phosphorylation and ROS generation. NADPH oxidase is the source of ROS in TX stimulated platelets. The proposed model helps explain why an incomplete inhibition of TP receptor results in residual platelet activation, and define new targets for antiplatelet treatment.

## Introduction

Thromboxane (TX) A_2_, or its synthetic and natural analogs have a critical role in platelet activation. These compounds, depending on the concentration used and their chemical structure, trigger different signaling pathways downstream the thromboxane-peroxide (TP) receptor that have been the object of several investigations. TP-α is the only functional receptor expressed in platelets and its capability to transduce intracellular signals depends on G protein coupling. Among Gα proteins coupled to TP-α, G13 α signals for shape change (Offermanns et al., 1994; Ohkubo et al., 1996; Klages et al., 1999; Zhang et al., 2009) whereas Gαq- also leads to platelet secretion and aggregation (Li et al., 2010a,b). The concentration of TP agonists is also a major determinant of the platelet response. Stimulation of washed platelets with the thromboxane TXA_2_ analog U46619 in the nanomolar range of concentrations, in the presence of the ADP scavenger apyrase and under non-aggregating conditions, was shown to induce both Rho kinase-induced phosphorylation of myosin light chain (MLC) and tyrosine phosphorylation signals; to note, these pathways were implicated in triggering of platelet shape change, but not secretion and aggregation (Minuz et al., 2006). However, platelets treated with low concentrations of U46619 in the presence of sub-stimulatory doses of epinephrine, that acts through a Gz coupled α2 adrenoreceptor, undergo a full release reaction and platelet aggregation response.

The use of specific inhibitors demonstrated that functional responses induced by U46619, as well as thrombin, require Src phosphorylation (Maeda et al., 1993; Gao et al., 2001; Senis et al., 2015). However, in response to thrombin analogs Src-family kinases activated via Gα13 were reported to inhibit Gαq-mediated increase in intracellular calcium and protein kinase C (PKC) activation, as well as platelet responses (Kim and Kunapuli, 2011). Additionally, a Src-dependent inhibitory role of Gα_13_ on activation of RhoA and platelet responses was also found in the context of integrin- dependent signaling (Gong et al., 2010). To add further complexity to the role of Src kinases in regulation of platelet responses several reports showed that tyrosine phosphorylation signals, calcium, PI3K and PKC may synergize in mediating Gαq-dependent platelet responses to thrombin (Li et al., 2010a,b; Bhavaraju et al., 2011; Xiang et al., 2012).

Crosstalk of Src with other pathways of platelet activation triggered by TXA_2_ analogs has not been extensively investigated. Calcium mobilization was implicated in the functional responses to platelet agonists and is indispensable for TP-dependent platelet aggregation (Berna-Erro et al., 2012). However, free intracellular calcium increase is very limited in response to low concentrations of U46619 or the partial TXA_2_ mimetic 8-iso-Prostaglandin (PG) F_2α_ (Minuz et al., 1998, 2002; Zhang et al., 2009). Moreover, it has been clearly demonstrated that some functional platelet responses occur via calcium-independent pathways (Paul et al., 1999; Huang et al., 2007). Reactive oxygen species (ROS) generation may certainly play a role as indicated by the evidence that platelet activation is blunted by antioxidants and NADPH oxidase inhibitors (Begonja et al., 2005; Tang et al., 2011; Violi and Pignatelli, 2014). Notably, ROS may be implicated in Src- and calcium-mediated signaling downstream G protein-coupled receptors. In fact, hydrogen peroxide was shown to induce Src activation (Inazu et al., 1990) and to be implicated in the activation of store-mediated calcium entry (SMCE) in human platelets (Rosado et al., 2004).

The present study aimed at defining the signaling pathway implicated in platelet activation induced by TXA_2_ analogs, further analyzing the role of Src kinases and the interplay of calcium and ROS in platelet activation to define the mechanisms responsible for their selectivity in eliciting different platelet functional responses.

## Materials and Methods

### Ethics Statement

The study protocol was approved by the Ethics Committee for Clinical Research of the Provinces of Verona and Rovigo. All subjects gave written informed consent in accordance with the Declaration of Helsinki.

#### Reagents

2-mercaptoethanol, Acetyl Salicylic Acid (ASA), apyrase VII, bromophenol blue, bovine serum albumin (BSA), citric acid, dextrose, cytochalasin B, EGTA, Hepes, KCl, NaCl, Na_2_HPO_4_, sodium citrate, SDS 20% and Tween-20 were supplied by Sigma Aldrich (Milan, Italy). Apocynin, epinephrine bitartrate (epinephrine, epi), Gö 6976, U46619, PP2 and Y27632 were supplied by Calbiochem (San Diego, CA, United States). Eptifibatide (Integrilin) was supplied by GlaxoSmithKline (Greenford, Middlesex, United Kingdom). EUK-134 and 8-iso-PGF_2α_, were obtained from Cayman Chemical (Ann Arbor, MI, United States). ECL^®^ reagent, HRP (horseradish peroxidase)-conjugated donkey anti-(rabbit Ig), goat antimouse IgG-HRP and Hybond C nitrocellulose were obtained by GE Healthcare Europe GmbH (Milan, Italy). Anti-phospho-Tyr (clone 4G10) were purchased from Millipore (Billerica, MA, United States). Anti phospho-Src family (Tyr416) and anti-Src (clone GD11) were purchased from Cell Signaling (Danvers, MA, United States). Anti phospho-Tyr PY99, anti-phospho MLC (myosin light chain; Thr18/Ser19) and anti MLC were purchased from Santa Cruz Biotechnology (Dallas, TX, United States). ADP and Collagen were supplied by Mascia Brunelli (Milan, Italy). Fura 2-AM and BAPTA/AM was obtained by Molecular Probes (Thermo Fisher Scientific, Waltham, MA, United States). PE-labeled CD62P-Selectin (clone CLBThromb/6) and IgG1 (Mouse) isotype were purchase from Beckman Coulter (Milan, Italy). FITC-labeled monoclonal antibody PAC-1 and FITC Mouse IgM, κ isotype control were from BD Biosciences (Milan, Italy). Dasatinib was purchased from Selleckchem (Munich, Germany).

#### Preparation of PRP for the *ex vivo* Analysis of Platelet Function

Platelet-rich plasma (PRP) was prepared from blood of patients treated with dasatinib and from blood of healthy volunteer, matched for age and sex, not taking any drugs in the previous 3 weeks.

Blood was taken 3 and 24 h after drug administration, corresponding to the expected peak of dasatinib in peripheral blood. Concerning the patients characteristics, the mean time from diagnosis of chronic myeloid leukemia (CML) in chronic phase was 25,5 months (range 6–60). All the patients discontinued treatment with imatinib due to adverse reaction or treatment failure and were on treatment with dasatinib (100 mg/day in four patients, 140 mg/day in one patient) from 9 months (range 2–32). In all subjects blood was drawn by venepuncture into 3,6 ml vacutainer (Venosafe, Terumo) with trisodium citrate 0.109 M. To block platelets cyclooxygenase activity ASA 100 μM was added to blood samples. For western blot experiments blood was collected in 3 ml Hirudine Blood Tube (Verum Diagnostica) in the presence of trisodium citrate 3.8%.

Platelet-rich plasma was obtained by centrifugation of blood at 200 × *g* at room temperature for 10 min and platelet count was estimated by an automated cell counter.

#### Preparation of Washed Platelets for the *in vitro* Experiments

For the *in vitro* experiments washed platelets were used. We used as anticoagulant an acid/citrate/dextrose mixture (sodium citrate14 mM citric acid, 11.8 mM and dextrose 18 mM) added with ASA 100 μM and apyrase VII 0.4 U mL^−1^. Washed platelets were obtained by centrifugation of blood samples at 200 × *g* for 10 min to obtain a PRP. This was followed by further centrifugation at 700 × *g* for 15 min at room temperature; platelets were then suspended in Hepes buffer (Hepes 10 mM, pH 7.4, NaCl 145 mM, KCl 5 mM, Na_2_HPO_4_ 0.5 mM and glucose 6 mM) in the presence of ASA 100 μM and apyrase VII 10 U mL^−1^. Platelet suspensions were kept at room temperature and tested within 2 h. When indicated, platelets were incubated (15 min at 37°C) with inhibitors/antioxidants before stimulation.

#### Immunoblot Analysis

Washed platelets (150 × 10^6^ platelets) were pre-incubated at 37°C, under static conditions, in the presence or absence of antioxidants/inhibitors as indicated in the Section “Results.” Then the platelets were stimulated for 40 s with specific agonists and in the presence of Ca^2+^ 1 mM. AFTER STIMULATION 4X SAMPLE BUFFER (TRIS/HCL 100 MM pH 6.8, 2-mercaptoethanol 200 mM, SDS 4%, glycerol 20% and Bromophenol Blue 0.4%) was added to the samples, which were then boiled for 3 min and stored at −80°C until use. Samples were separated by SDS/PAGE as previously described (Minuz et al., 2006). For western blot analysis in the *ex vivo* experiments PRP was stimulated for 40 s at 37°C with agonists in the presence of apyrase VII 10 U mL^−1^ and eptifibatide 10 μg mL^−1^. The reaction was stopped with the addition of Hepes buffer (2 ml) and after centrifugation at 10000 × *g* for 10 s, platelets were lysed in 4X sample buffer at 95°C and treated as described before.

#### Platelet Free Intracellular Calcium [Ca^2+^]i

[Ca^2+^]i was measured in washed platelets with the fluorescence indicator Fura 2-AM, according to the method described by Pollock et al. (1986).

Washed platelets suspended in HEPES buffer (4 × 10^8^ cells mL^−1^), in the presence of apyrase VII 10 U mL^−1^ and eptifibatide 10 μg mL^−1^, were loaded with Fura 2-AM 2 μM for 25 min at 32°C. After centrifugation at 200 × *g* for 10 min 40 × 10^6^ platelets were placed in quartz cuvettes and fluorescence measurements were carried out at 37°C using a QuantaMaster spectrofluorometer (PTI, Japan) with magnetic stirring. After platelets stimulation the fluorescence signal was monitored by using double excitation wavelength of 340 and 380 nm and an emission wavelength of 510 nm. [Ca^2+^]i was expressed as concentration using a Kd for Fura-2 of 226 nM; the calculations were done according to the equations of Grynkiewicz et al. (1985).

#### Platelet Aggregation

For *in vitro* aggregation washed platelets (300.000 cells μL^−1^) were pre treated with antioxidants/inhibitors at 37°C for 10 min in the presence of apyrase VII 10 U mL^−1^ and ASA 100 μM. Platelets were transferred into cuvettes and incubated with CaCl_2_ 1 mM and MgSO_4_ 1 mM for 1 min at 37°C under continuous stirring at 1000 rpm. Platelet aggregation was monitored for 5 min after the addition of the agonist, using the Born’s turbidimetric method in a four channel aggregometer (APACT 4004, Labitech and Chrono-log Model 700 Whole Blood/Optical Lumi-Aggregometer, Chrono-Log Corp.). The rate of platelet aggregation was calculated as change in percentage of transmitted light (%T), according to Born and Cross (1963). For the *ex vivo* experiments, aggregation tests were performed using PRP in the presence/absence of apyrase VII 10 U mL^−1^ with platelet count adjusted at 300.000 platelets μL^−1^.

#### Flow-Cytometry Analysis of Platelet a Granule Secretion and α_IIb_β_3_ Activation

For the *in vitro* and *ex vivo* experiments, washed platelet suspension (50.000 platelets μL^−1^) was incubated at 37°C for 15–20 min with or without antioxidants/inhibitors as indicated in the Section “Results.” PE-labeled anti-CD62 (P-Selectin) and FITC-labeled monoclonal antibody PAC-1, a ligand mimetic monoclonal antibody that specifically binds to the active form of integrin α_IIb_β_3_ complex, were added to platelet suspensions before the agonists at room temperature. In all the experiments two isotype-matched irrelevant mouse IgG1 FITC and PE labeled were included as negative control. After incubation at room temperature, samples were diluted in PBS and analyzed by flow cytometry (Cytomics FC 500; Beckman Coulter), using dual color fluorescence. Platelets were identified on the basis of their Forward Scatter and Side Scatter properties.

For the *in vitro* and *ex vivo* experiments, the expression of P-selectin and the activation of the fibrinogen receptor with PAC-1 were studied in PRP diluted at 20.000 platelets μL^−1^ in presence of ASA 100 μM and in the presence or absence of apyrase VII 10 U mL^−1^.

#### Measurement of ROS

Reactive oxygen species generation was measured using a commercial kit according to the manufacturer’s instruction (Total ROS/Superoxide Detection Kit, Enzo Life Sciences). Briefly, washed platelets (100.000 μL^−1^) were pre-treated, at 37°C for 15 min, in the absence or presence of apyrase VII 10 Um L^−1^ with apocynin 300 μM and BAPTA/AM 20 μM according to the experimental procedures. Platelets were collected by centrifugation at 400 × *g* for 5 min, incubated with 1 μM ROS/Superoxide detection mix for 60 min at 37°C in the presence or absence of the agonist. Changes in the fluorescence intensity were measured using a microplate fluorescence reader (Victor X, PerkinElmer) at excitation/emission wavelengths of 488/520 nm.

In each of the previously described experimental sets all the tested conditions were analyzed using platelet preparations obtained from a single blood samples, replicates were always from different blood donors.

### Statistics

For statistical analysis, all data were analyzed with GraphPad Prism software v.5.03 (GraphPad Software, San Diego, CA, United States). Data are presented as Mean and Standard Error in figures or Mean and Standard deviation in tables. Data concerning all the parameters included in each experimental set were obtained from the analysis of a single platelet preparation (n subjects). This allowed to perform the statistical analysis for multiple comparisons using the One-way ANOVA with *post hoc* pairwise comparisons performed using Newman–Keuls or Dunnett’s test, as indicated in individual tables and figure legends, or the Two-way ANOVA followed by Bonferroni’s test, as indicated in figure legends. *P*-value < 0.05 was assumed as statistically significant.

## Results

### Functional Role of Tyrosine Kinases on Platelet Responses to Soluble Agonists: *In vitro* and *ex vivo* Study

Based on our previous studies implicating tyrosine phosphorylation signals in platelet responses to a combination of low doses of a thromboxane analog and sub-stimulatory doses of epinephrine (Minuz et al., 2006), we addressed whether a Src-family kinase inhibitor affected platelet function *in vitro* and *ex vivo*. Using washed platelets in the presence of the ADP scavenger apyrase and ASA we found that the specific inhibitor PP2 inhibited both α granule secretion (CD62P expression %) and the expression of the active form of the fibrinogen receptor (PAC-1 binding %) in response to low doses of U46619 plus epinephrine (**Table 1**). In contrast, the response to optimal, stimulatory doses of U46619 was only marginally affected.

**Table 1:** Effects of the Src-family inhibitor PP2 on the expression of P-Selectin (CD62) and the active form of the fibrinogen receptor, as assessed using the monoclonal antibody PAC-1.

Conditions	Active fibrinogen receptor	CD62P
Resting (*n* = 3)	0.7 ± 0.6	1.6 ± 1.3
+ U46619 1 μM	7.8 ± 5.4	14.0 ± 4.5^∗^
+ U46619 1 μM + PP2 10 μM	5.2 ± 6.1	12.3 ± 7.5
Resting (*n* = 5)	1.6 ± 1.9	1.9 ± 1.1
+ U46619 50 nM + Epinephrine 10 μM	26.3 ± 13.1^∗†^	26.6 ± 9.2^∗†^
+ U46619 50 nM + Epinephrine 10 μM + PP2 10 μM	11.3 ± 5.8^∗†^	8.6 ± 3.1^∗†^

*Washed platelets were stimulated by U46619 1 μM and U46619 50 nM with epinephrine 10 μM in the presence of ASA 100 μM and apyrase VII 10 U mL^−1^. Data are expressed as % of positive events. Repeated-measures One-way ANOVA, followed by Newman–Keuls as post hoc test was applied to compare each agonist and inhibitors with resting conditions. Mean ± SD, ^∗^P < 0.05 vs. resting platelets, ^†^P < 0.05 stimulated –PP2 vs. stimulated +PP2.*

**FIGURE 1:**
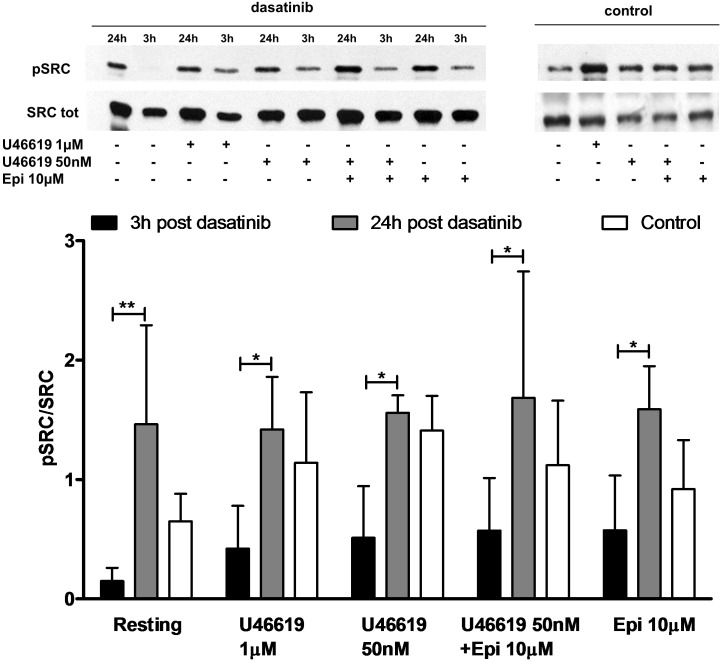
*Ex vivo* effect of dasatinib on Src phosphorylation. PRP was isolated in the presence of ASA 100 μM and apyrase VII 10 U mL^−*1*^ from health volunteer (*n* = 7) or from patient (*n* = 5) 3 and 24 h post treatment with dasatinib. PRP was stimulated by U466191 μM, U46619 50 nM, epinephrine (epi) 10 μM, U46619 50 nM plus epinephrine 10 μM. Two-way ANOVA followed by Bonferroni’s test was applied to compare platelet response to each agonist with resting conditions in the presence or absence of dasatinib. Data are expressed as Mean ± SE, ^∗^*P* < 0.05, ^∗∗^*P* < 0.01.

The role of a tyrosine phosphorylation pathways in this response was further investigated examining platelets obtained from patients affected by CML treated with dasatinib, an Abl/Src dual specificity inhibitor. We found that in platelets collected 3 h after the oral administration of dasatinib, the constitutive phosphorylation of Src was markedly lower than in platelets collected 24 h after drug administration (**Figure 1**). Examining platelet responses in PRP we detected a robust increase in P selectin expression (CD 62) and α_IIb_β_3_ activation when U46619 1 μM or U46619 50 nM in combination with epinephrine 10 μM were used (**Table 2**). Notably, these responses were significantly lower in platelets from CML patients after 3 h from administering dasatinib and went back to pre-treatment levels after 24 h. Reductions in constitutive Src phosphorylation, P-selectin expression and α_IIb_β_3_ activation after 3 h from dasatinib treatment correlated with a decreased aggregation response in response to U46619 1 μM or U46619 50 nM in combination with epinephrine 10 μM (**Table 3**). The effects of collagen and ADP are shown for comparison.

**Table 2:** *Ex vivo* effect of dasatinib on the expression of P-Selectin (CD62%) and the active form of fibrinogen receptor (%).

	CD62			Active fibrinogen receptor		
Conditions	Control *n* = 7	3 h post Dasatinib *n* = 5	24 h post Dasatinib *n* = 5	Control *n* = 7	3 h post Dasatinib *n* = 5	24 h post Dasatinib *n* = 5
**Apyrase 10 U mL^−1^**						
Resting	1.2 ± 1.1	0.4 ± 0.3	1.4 ± 1.0	0.4 ± 0.1	0.3 ± 0.1^∗^	0.2 ± 0.1^∗^
U46619 1 μM	9.0 ± 3.3	5.5 ± 6.7	17.3 ± 12.5	5.8 ± 3.4	2.0 ± 2.4^∗^	10.3 ± 9.4
U46619 50 nM	1.9 ± 1.6	0.4 ± 0.4	4.5 ± 4.7^∗^	0.6 ± 0.2	0.2 ± 0.1^∗^	1.1 ± 1.7
Epinephrine 10 μM	6.7 ± 3.9	1.3 ± 1.7^∗†^	8.4 ± 4.3^∗^	3.3 ± 2.4	0.4 ± 0.4^∗^	3.9 ± 3.1^∗^
U46619 50 nM + epinephrine 10 μM	24.5 ± 16.6	7.4 ± 9.4	24.5 ± 23.1	14.3 ± 11.1	1.8 ± 2.3^∗^	15.8 ± 16.0
U46619 1 μM + eptifibatide 10 μg mL^−1^	22.7 ± 10.9	11.6 ± 11.8	28.0 ± 17.4	0.4 ± 0.2	0.6 ± 0.8	0.2 ± 0.1^∗^
ADP 5 μM	2.2 ± 1.6	0.9 ± 0.9^†^	2.7 ± 1.3	0.5 ± 0.1	0.2 ± 0.1^†^	0.5 ± 0.4
Collagen 10 μg mL^−1^	2.4 ± 0.9	1.6 ± 1.3	2.9 ± 0.8	1.5 ± 0.5	1.3 ± 0.6	1.5 ± 0.6
Collagen 10 μg mL^−1^ + U46619 50 nM	3.9 ± 0.9	2.5 ± 1.6	4.7 ± 2.8	6.0 ± 9.6	1.5 ± 0.6	3.0 ± 1.7
**Without apyrase**						
Resting	1.7 ± 1.4	0.2 ± 0.1^∗^	1.7 ± 1.4	0.4 ± 0.2	0.2 ± 0.1	0.2 ± 0.1
U46619 1 μM	31.5 ± 19.2	11.0 ± 12.4^†^	39.2 ± 31.4	29.7 ± 24.7	3.9 ± 5.0^∗†^	33.6 ± 27.7
U46619 50 nM	2.5 ± 1.5	0.4 ± 0.2^∗†^	6.36 ± 7.7^∗^	0.8 ± 0.5	0.2 ± 0.1^∗^	2.6 ± 3.9
Epinephrine 10 μM	8.2 ± 3.6	1.2 ± 1.4^∗†^	10.5 ± 5.8	6.6 ± 3.9	0.6 ± 0.6^∗†^	7.9 ± 5.9
U46619 50 nM + epinephrine 10 μM	32.2 ± 20.8	9.2 ± 11.4^†^	32.0 ± 25.6	24.4 ± 15.7	2.5 ± 3.1^∗†^	27.3 ± 25.3
U46619 1 μM + eptifibatide 10 μg mL^−1^	64.2 ± 16.7	24.4 ± 26.5^∗†^	47.6 ± 34.9^∗^	0.2 ± 0.1	0.2 ± 0.1	0.1 ± 0.1
ADP 5 μM	40.5 ± 8.1	21.1 ± 20.9^†^	41.4 ± 18.1	49.7 ± 16.7	20.5 ± 19.4^∗†^	46.7 ± 25.5
Collagen 10 μg mL^−1^	2.6 ± 0.9	1.5 ± 0.9^†^	3.4 ± 1.0	1.6 ± 0.6	1.3 ± 0.5	1.8 ± 0.7
Collagen 10 μg mL^−1^ + U46619 50 nM	4.1 ± 0.6	2.3 ± 1.2^∗†^	4.6 ± 2.9	2.7 ± 1.1	1.5 ± 0.4^†^	3.6 ± 1.6

*Mean ± SD, ^∗^P < 0.05: vs. control, ^†^P < 0.05 3 h post dasatinib vs. 24 h post dasatinib. PRP was prepared in the presence of ASA 100 μM with and without apyrase VII 10 U mL^–1^ from healthy volunteers or patients, 3 and 24 h post treatment with dasatinib, in the presence or absence of eptifibatide 10 μg mL^–1^. Data are expressed as % of positive events. One-way ANOVA, followed by Newman–Keuls as post hoc test was applied to compare each agonist in the presence or absence of dasatinib with resting conditions in patients and controls. Active fibrinogen receptor and CD62 were separately analyzed.*

**Table 3:** Effect of dasatinib on platelet aggregation (% aggregation at 5 min).

Conditions	Control	3 h post Dasatinib	24 h post Dasatinib
**Apyrase 10 U mL^−1^**			
U46619 1 μM	57.9 ± 7.7	21.4 ± 20.7^∗†^	51.4 ± 27.7
Epinephrine 10 μM	27.4 ± 6.4	7.9 ± 8.8^∗†^	30.8 ± 11.2
U46619 50 nM + Epinephrine 10 μM	30.9 ± 10.0	18.6 ± 25.6	31.7 ± 10.1
Collagen 10 μg mL^−1^	17.4 ± 7.1	6.1 ± 1.6^∗†^	18.9 ± 8.9
ADP 5 μM	0.0 ± 0.0	1.3 ± 2.2	0.2 ± 0.5
Collagen 10 μg mL^−1^ + U46619 50 nM	19.8 ± 9.2	3.5 ± 2.5^∗^	19.8 ± 15.6
**Without apyrase**			
U46619 1 μM	81.2 ± 9.0	15.9 ± 28.6^∗^	62.5 ± 32.3
Epinephrine 10 μM	25.5 ± 5.5	7.9 ± 5.6^∗†^	26.2 ± 8.5
U46619 50 nM + Epinephrine 10 μM	35.5 ± 18.6	17.9 ± 23.7	32.2 ± 12.7
Collagen 10 μg mL^−1^	74.9 ± 11.8	10.5 ± 15.1^∗†^	66.4 ± 12.2
ADP 5 μM	76.2 ± 6.3	31.6 ± 30.6^∗^	69,1 ± 9.1
Collagen 10 μg mL^−1^ + U46619 50 nM	74.8 ± 16.9	9.8 ± 13.9^∗†^	63.7 ± 13.5

*Mean ± SD, n = 5, ^∗^P < 0.05 vs. control, ^†^P < 0.05 3 h post dasatinib vs. 24 h post dasatinib. PRP of patients/health volunteer was stimulated in the presence or absence of apyrase VII 10 U mL^−1^ and ASA 100 μM, respectively, 3 and 24 h post dasatinib administration. One-way ANOVA, followed by Newman–Keuls as post hoc test was applied to compare each agonist with or without inhibitor and controls.*

### Calcium Mobilization in Response to Thromboxane A_2_ Analogs

**FIGURE 2:**
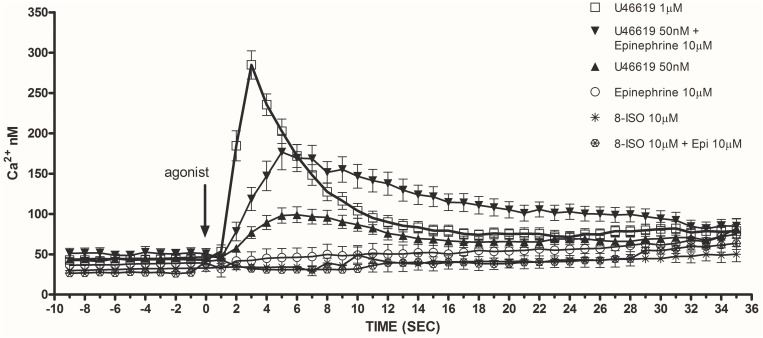
Maximal release of intracellular calcium in washed and non-aggregating platelets (in presence of ASA 100 μM, apyrase VII 10 U mL^−1^, eptifibatide 10 μg mL^−1^) stimulated by U46619 1 μM, U46619 50 nM, U46619 50 nM + epinephrine (epi) 10 μM, epi alone 10 μM, 8-iso PGF2α 10 μM, 8-iso PGF2α 10 μM + epi 10 μM (Ca^2+^ nM). Repeated-measures Two-Way ANOVA was applied to compare the effects of platelets agonists at different time points. Significant differences (*P* < 0.0001) were observed comparing U46619 1 mmol/L with all the other agonists, except U46619 50 nM + epi 10 μM. Data are expressed as Mean ± SE.

**FIGURE 3:**
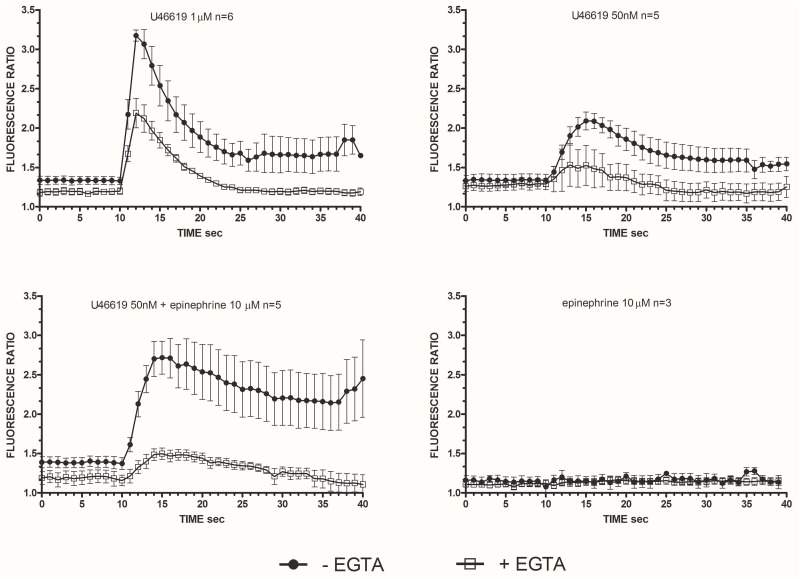
Intracellular Ca^2+^ release in stimulated washed platelets in presence/absence of extracellular calcium. Washed platelets in non-aggregating condition (eptifibatide 10 μg mL^−1^, ASA 100 μM, apyrase VII 10 U mL^−1^) were stimulated by U46619 1 μM, U46619 50 nM, U46619 50nM + epinephrine 10 μM, and epinephrine 10 μM. Fluorescent emission at 510 nm was measured induced by double excitation wavelength of 340 and 380 nm. The ratio of the two fluorescence is proportional to the concentration of the released intracellular calcium. Repeated-measures Two-Way ANOVA was applied to compare the effects of platelets agonists at different time points. Significant differences (*P* < 0.0001) were observed comparing intracellular Ca^2+^ release induced by U46619, but not epinephrine (*P* = n.s.), with and without EGTA. Data are expressed as Mean ± SE.

Considering that calcium signals play an indispensable role in platelet activation (Varga-Szabo et al., 2009) we addressed whether the different doses of the TXA_2_ analog U46619, tested alone or with epinephrine, which we found to elicit a tyrosine phosphorylation response, were also able to trigger calcium mobilization (**Figure 2**). Experiments were performed in the presence of ASA, apyrase, and eptifibatide using washed platelets under stirring. Compared to doses of 1 μM, U46619 50 nM triggered only a modest increase of calcium transients (peak value increment of about 260 nM for 1 μM U46619 and 60 nM for U46619 50 nM). However, when added in combination with epinephrine 10 μM, that *per se* was unable to trigger any increase in cytosolic calcium, U46619 50 nM triggered a net calcium response (peak value increment about 140 nM) that was more prolonged than that induced by U46619 1 μM. The increase in free intracellular calcium induced by low doses U46619 alone or in combination with epinephrine required calcium influx, since it was almost abolished in the presence of EGTA, while calcium increase induced by U46619 1 μM was attenuated, but not abolished by EGTA (**Figure 3**). To further explore the selectivity of calcium signals downstream the TP receptor, we tested the effects of 8-iso-PGF_2α_ 10 μM under the same experimental conditions. However, either alone or in combination with epinephrine, 8-iso-PGF_2α_ failed to elicit any calcium response (**Figure 2**).

Calcium mobilization was not altered by any of the tested antagonists of specific pathways: the Src kinase inhibitor PP2, ROS scavenger NADPH oxidase inhibitor apocynin, Rho kinase inhibitor Y27632, the protein kinase C inhibitor Gö 6976 (**Table 4**).

**Table 4:** Maximal release of intracellular calcium (expressed in nM) in washed platelets (in the presence of ASA 100 μM, apyrase 10 U mL^−1^, eptifibatide 10 μg mL^−1^) stimulated by U46619 1 μM, U46619 50 nM, U46619 50 nM + epinephrine 10 μM, epinephrine 10 μM, 8-iso-PGF_2α_ 10 μM, 8-iso-PGF_2α_ 10 μM + epinephrine 10 μM.

Conditions	Resting	U46619 1 μM	U46619 50 nM	U46619 50 nM + Epi 10 μM	Epi 10 μM
Platelets (*n* = 15)	43.4 ± 22.8	298.9 ± 91.8^∗^	104.8 ± 43.3^∗^	185.4 ± 69.5^∗†^	53.7 ± 35.1
+ Apocynin 300 μM (*n* = 6)	49.6 ± 11.9	276.3 ± 70.9^∗^	119.2 ± 46.3^∗^	175.1 ± 59.9^∗^	77.1 ± 4.6
+ EUK-134 250 μM (*n* = 6)	58.4 ± 8.9	233.7 ± 43.8^∗^	115.6 ± 15.1^∗^	181.1 ± 46.6^∗†^	78.8 ± 26.3
+ PP2 10 μM (*n* = 3)	63.6 ± 7.9	317.1 ± 80.1^∗^	148.5 ± 49.0^∗^	203.9 ± 47.2^∗^	88.7 ± 15.3
+ Y27632 30 μM (*n* = 3)	65.0 ± 16.1	293.9 ± 23.7^∗^	134.8 ± 43.2^∗^	179.0 ± 54.4^∗^	139.3 ± 2.4^∗^
+ Cytochalasin B 20 μM (*n* = 3)	18.0 ± 4.27	226.1 ± 9.2^∗^	69.4 ± 15.6	–	52.4 ± 9.7
+ Gö 6976 (*n* = 3)	48.8 ± 25.3	301.8 ± 85.4^∗^	51.0 ± 38.5	239.8 ± 22.6^∗†^	46.2 ± 36.5

	**Resting**		**8-isoPGF_2α_ 10 μM**	**8-isoPGF_2α_ 10 μM + Epi 10 μM**	

Platelets (*n* = 3)	30.5 ± 4.4		62.0 ± 9.9^∗^	74.6 ± 13.4^∗^	

*Mean ± SD, ^∗^P < 0.05 resting vs. activated. ^†^P < 0.05 U46619 50 nM + epinephrine 10 μM vs. U46619 50 nM and epinephrine 10 μM. One-way ANOVA, followed by Newman–Keuls as post hoc test was applied to compare each agonist in the presence or absence of inhibitors with resting conditions.*

### Cross-Talk Between Calcium and Tyrosine Phosphorylation Signals in Platelet Activation

**FIGURE 4:**
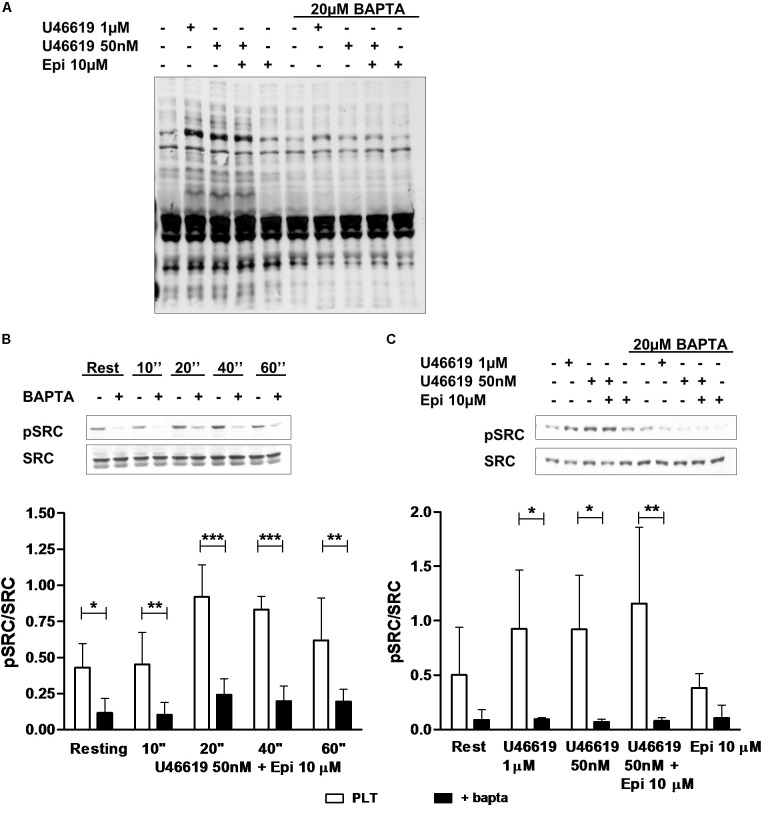
Western Blotting analysis of tyrosine **(A)** and Src **(B,C)** phosphorylation. Washed platelets were isolated in non-aggregating condition (eptifibatide 10 μg mL^−1^, ASA 100 μM, apyrase VII 10 U mL^−1^) and stimulated with agonists. The reaction was stopped by adding sample buffer 4× at 100°C. **(B)** Typical pattern of Src phosphorylation after stimulation of 10, 20, 40, and 60 s with U46619 50 nM + epi 10 μM in condition of intracellular calcium-presence or absence. Graphic is representative of four experiment. **(C)** Typical pattern of Src phosphorylation after stimulation of 40 s with U46619 1 μM, U46619 50 nM, U46619 50 nM + epi 10 μM, and epi 10 μM in presence or absence of BAPTA 20 μM. Graphic shows five experiments. Repeated-measures Two-Way ANOVA was applied to compare the effects of platelets agonists on Src phosphorylation at different time points in the presence of different agonists **(B,C)**. Mean ± SE, ^∗^*P* < 0.05, ^∗∗^*P* < 0.01, ^∗∗∗^*P* < 0.001.

In order to understand whether tyrosine phosphorylation triggered by low U46619 doses (Minuz et al., 2006) was distinct or downstream calcium signals, we examined total protein tyrosine phosphorylation in control platelets or platelets loaded with the calcium-chelator BAPTA. As reported in **Figure 4A**, U46619 50 nM was as potent as 1 μM in increasing phosphorylation of tyrosine residues. However, loading of platelets with BAPTA that blunted increase in intracellular calcium (not shown), decreased U46619-stimulated tyrosine phosphorylation to background levels. Similar results were obtained examining Src tyrosine phosphorylation in response to U46619 50 nM in combination with epinephrine 10 μM in time-course experiments (**Figure 4B**) and U46619 1 μM or U46619 50 nM alone and in combination with epinephrine 10 μM (**Figure 4C**). We conclude that increase in intracellular calcium precedes and regulates tyrosine phosphorylation signals in platelets stimulated with U46619 alone or in combination with epinephrine.

### Role of Changes in Free Intracellular Calcium in Functional Platelet Responses

**FIGURE 5:**
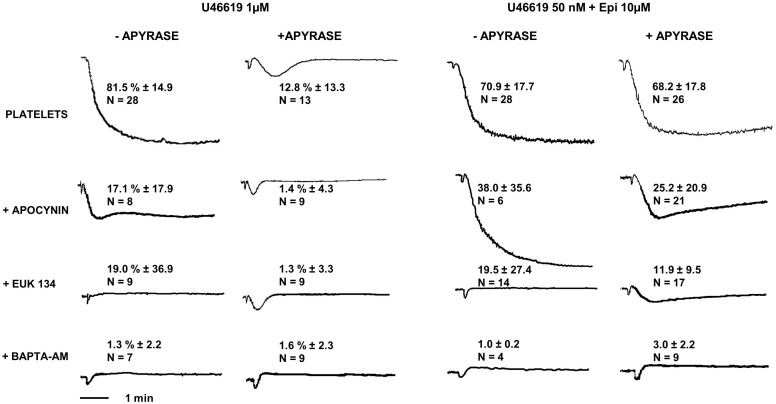
Platelet aggregation induced by U46619 1 μM and U46619 50 nM + epi 10 μM. Washed platelets in presence or absence of apyrase VII were pre-treated with the NADPH oxidase inhibitor apocynin 300 μM, the reactive oxygen species (ROS) scavenger EUK-134 250 μM or the intracellular calcium chelator BAPTA-AM 20 μM, respectively. Data are expressed as Mean ± SE.

In order to understand whether an increase in intracellular free calcium plays a critical role also in triggering platelet responses, we addressed the expression of the active form of the fibrinogen receptor, secretion of α granules, platelet shape change and aggregation in platelets loaded with BAPTA and stimulated with U46619 alone or in combination with epinephrine. As shown in **Table 5**, loading of platelets with BAPTA decreased both expression of an activation epitope in α_IIb_β_3_ and the degranulation response in response to a combination of low U46619 doses and epinephrine to background levels. Blunting the calcium increase also resulted in inhibition of the response to optimal (1 μM) doses of U46619. Similar results were obtained examining platelet aggregation (**Figure 5**). We conclude that calcium signals play an essential role in regulating both tyrosine phosphorylation and functional responses in platelets.

**Table 5:** Effects of calcium chelators BAPTA-AM 20 μM (intracellular calcium chelator) or EGTA 1 mM (extracellular calcium chelator) on the expression of the active fibrinogen receptor and P-Selectin (CD62) in washed platelets stimulated with U46619 1 μM or U46619 50 nM + epinephrine (epi) 10 μM.

	Active fibrinogen receptor		CD62+	
Conditions	With apyrase	Without apyrase	With apyrase	Without apyrase
Resting	0.83 ± 0.49 *n* = 8	1.10 ± 0.63 *n* = 10	2.03 ± 1.37 *n* = 6	3.46 ± 0.88 *n* = 4
+ BAPTA 20 μM	0.67 ± 0.37 *n* = 7	0.64 ± 0.22 *n* = 5	0.20 ± 0.21 *n* = 5^∗^	0.26 ± 0.23 *n* = 5^∗^
U46619 50 nM + Epi 10 μM	16.70 ± 20.27 *n* = 9^∗^	29.66 ± 23.41 *n* = 9^∗^	12.48 ± 6.21 *n* = 6^∗^	26.28 ± 13.47 *n* = 6^∗^
U46619 50 nM + Epi 10μM + BAPTA 20 μM	0.71 ± 0.35 *n* = 9^†^	3.49 ± 7.61 *n* = 8^†^	0.87 ± 0.92 *n* = 6^†^	0.71 ± 0.59 *n* = 7^∗†^
U46619 50 nM + Epi 10 μM + EGTA	1.02 ± 1.05 *n* = 6	1.80 ± 2.16 *n* = 6^†^	13.73 ± 9.74 *n* = 6	27.51 ± 15.25 *n* = 6
U46619 1 μM	1.38 ± 0.49 *n* = 8^∗^	20.85 ± 23.83 *n* = 6^∗^	3.80 ± 1.85 *n* = 6	18.54 ± 7.37 *n* = 6^∗^
U46619 1 μM + BAPTA 20 μM	0.63 ± 0.41 *n* = 8^†^	0.74 ± 0.28 *n* = 6	1.00 ± 0.96 *n* = 5^†^	0.78 ± 0.67 *n* = 6^†^
U46619 1 μM + EGTA	0.73 ± 0.55 *n* = 6^†^	0.86 ± 0.49 *n* = 6	4.16 ± 2.00 *n* = 6	23.41 ± 15.45 *n* = 6

*Mean ± SD, ^∗^P < 0.05 stimulated vs. resting platelets, ^†^P < 0.05 stimulated + BAPTA/EGTA vs. stimulated. In the presence of ASA 100 μM, eptifibatide 10 μg mL^−1^, with and without apyrase 10 U mL^−1^. Data are expressed as % of positive events. One-way ANOVA, followed by Newman–Keuls as post hoc test was applied to compare each agonist in the presence or absence of inhibitors with resting conditions. Active fibrinogen receptor and CD62 were separately analyzed.*

### ROS Generation in Platelets Stimulated With U44619

Considering that TP stimulation with U46619 was reported to induce ROS generation in a dose-dependent manner (Begonja et al., 2005; Wilson et al., 2009), we addressed whether ROS are implicated in the platelet response to low U46619 doses in combination with epinephrine using two different inhibitors of ROS generation (Dharmarajah et al., 2010). We found that both apocynin and EUK-134 strongly inhibited platelet aggregation induced by both optimal dose of U46619 and a combination of low U46619 doses and epinephrine (**Figure 5**). Additionally, these compounds inhibited both the expression of an activation epitope in α_IIb_β_3_ and the degranulation response (**Table 6**).

**Table 6:** Effect of the NADPH oxidase inhibitor apocynin 300 μM and the ROS scavenger EUK-134 250 μM on the expression of P-Selectin (CD62P) and of active form of fibrinogen receptor in washed platelets pre-treated with ASA 100 μM and apyrase VII 10 U mL^−1^.

Conditions	CD62	Active fibrinogen receptor
Resting	2.08 ± 1.26 *n* = 12	0.72 ± 0.43 *n* = 12
+ Apocynin 300 μM	1.09 ± 0.10 *n* = 4	0.35 ± 0.13 *n* = 4
+ EUK-134 250 μM	0.87 ± 0.11 *n* = 4	0.30 ± 0.11 *n* = 4
U46619 1 μM	6.97 ± 2.88 *n* = 6^∗∗∗^	0.97 ± 0.56 *n* = 6
+ Apocynin 300 μM	3.18 ± 0.89 *n* = 5^†^	0.48 ± 0.17 *n* = 5
+ EUK-134 250 μM	1.71 ± 0.44 *n* = 4^†^	1.49 ± 0.17 *n* = 4
U46619 50 nM + epi 10 μM	16.47 ± 9.90 *n* = 10^∗∗∗^	9.14 ± 9.24 *n* = 10^∗^
+ Apocynin 300 μM	9.20 ± 4.84 *n* = 6^‡^	2.43 ± 2.00 *n* = 6^∗^
+ EUK-134 250 μM	6.06 ± 7.40 *n* = 11^‡^	3.63 ± 3.61 *n* = 11^∗^

*Mean ± SD, ^∗∗∗^P < 0.0001 vs. resting, ^∗^P < 0.05 vs. resting, ^†^P < 0.0001 vs. U46619, 1 μM, ^‡^P < 0.05 vs. U46619 50 nM + epinephrine 10 μM. Data are expressed as % of positive events. One-way ANOVA, followed by Newman–Keuls as post hoc test was applied to compare each agonist in the presence or absence of inhibitors with resting conditions. Active fibrinogen receptor and CD62 were separately analyzed.*

**FIGURE 6:**
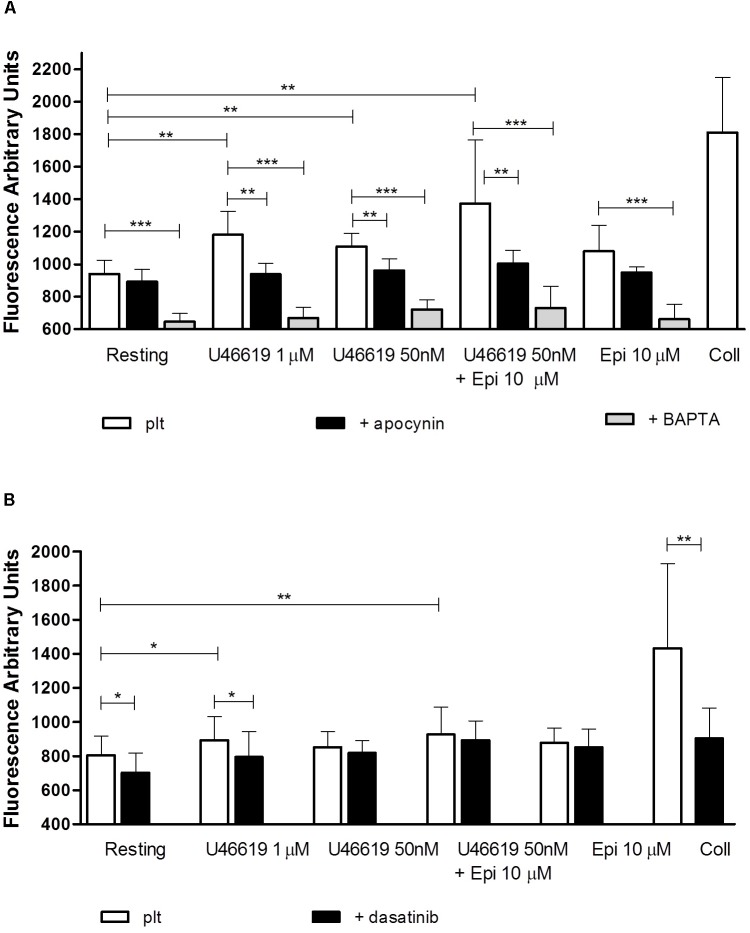
Reactive oxygen species generation (measured as Fluorescence Arbitrary Units-FUA) induced by agonists in washed platelets in presence of apyrase VII 10 U mL^−1^, ASA 100 μM and eptifibatide 10 μg mL^−1^ **(A,B)**. Platelets were pre-treated with the NADPH oxidase inhibitor apocynin 300 μM, the intracellular calcium chelator BAPTA-AM 20 μM **(A)**, or the tyrosine kinase inhibitor dasatinib 150 nM **(B)**. Repeated-measures One-way ANOVA, with the *Newman–Keuls* test for *post hoc* analysis were applied to compare independently each platelet agonist in the presence or absence of inhibitors with resting conditions. Data are expressed as Mean ± SE. Indicates the signficance level: ^∗^*P* < 0.05, ^∗∗^*P* < 0.01, ^∗∗∗^*P* < 0.0001.

In order to know whether in our assay conditions platelets were able to generate ROS we assayed ROS generation in response to U46619 either alone or in combination with epinephrine. As reported in **Figure 6A**, U46619 50 nM triggered a limited increase of ROS generation. While epinephrine alone did not trigger any significant increase in ROS generation, this was observed when epinephrine was used in combination with U46619 50 nM or when U46619 1 μM was used as a stimulus (*P* < 0.001 and *P* < 0.01, respectively, applying One way ANOVA followed by Dunnett’s test to analyze cumulatively data from the two sets of experiments shown in **Figures 6A,B**). Notably, ROS generation was blunted in platelets loaded with the calcium chelator BAPTA, thus suggesting that calcium signals are located upstream the activation NADPH oxidase. To investigate the relations between Src phosphorylation and ROS generation, we analyzed the effects of the specific dual Abl/Src inhibitor dasatinib, added *in vitro* to washed platelets that subsequently stimulated with agonists (PP2 could not be used, since interfering with fluorescence emission). In the presence of dasatinib, limited reduction in ROS generation was observed under basal conditions and after stimulation with 1 μM U46619, but not when 50 nM U46619 was tested alone or in combination with 10 μM epinephrine (**Figure 6B**). By comparison, collagen 10 μg mL^−1^, which was the strongest stimulus for platelet ROS generation in our experimental condition (**Figures 6A,B**), when tested in washed platelets pretreated with dasatinib reduced ROS generation by approximately 40% (**Figure 6B**). Cumulating the data from the two sets of experiments (*n* = 12), we observed that only 1 μM U46619 and 50 nM U46619 plus 10 μM epinephrine induced significant increase in ROS generation (*P* < 0.001 and *P* < 0.01, respectively, by One way ANOVA followed by Donnett’s *post hoc* test).

## Discussion

The major new finding from the present study is the requirement of calcium signals both for Src tyrosine kinase activation and NOX dependent ROS-generation downstream TP receptor. These three signals induce platelet secretion and aggregation when platelets are stimulated by low concentrations of U46619 and epinephrine. Quantitatively different increases in free intracellular calcium concentrations differentiate platelet responses to U46619.

Stimulation of washed platelets with low concentration (50 nM) of U46619 was previously shown to elicit MLC phosphorylation through a signaling pathway that requires Src and Rho-Rho kinase to induce platelet shape change (Li et al., 2010a). In the presence of epinephrine 10 μM plus U46619 50 nM, both release reaction and platelet aggregation were induced (Minuz et al., 2006). As confirmed by the present investigation, these events occur independently of released ADP. In fact, both the activation of the fibrinogen receptor and platelet aggregation, but not platelet secretion, induced by 1 μM U46619 are blunted in the presence of 10 U mL^−1^ apyrase, a scavenger of ADP, while platelet response to 50 nM U46619 plus 10 μM epinephrine is not altered by apyrase (see **Tables 2**, **3**) (Minuz et al., 2006). Therefore, all the experiments exploring the TXA_2_ signaling pathways were performed in the presence of apyrase 10 U mL^−1^.

We confirm the role of Src in platelet secretion performing our experiments in the absence of any signal ensuing from released ADP and under non-aggregating conditions. In fact, both dasatinib *ex vivo* (**Table 2**) and PP2 *in vitro* (**Table 1**) blunted P-selectin expression also when activation of the fibrinogen receptor was prevented by eptifibatide (Li et al., 2010b). Under the same experimental conditions, tyrosine kinase signaling also modulates the activation of α_IIb_β_3_ and platelet aggregation (**Tables 2**, **3**), as previously observed (Minuz et al., 2006; Li et al., 2010a). This finding is partly at variance with the previous observation that dasatinib does not inhibit P-selectin expression in thrombin or ADP-simulated platelets (Gratacap et al., 2009).

Src phosphorylation is an early event in the pathways operated by TXA_2_ analogs. To locate Scr activation, we investigated calcium signals. We found that an increase in intracellular calcium is necessarily required for functional responses and Src phosphorylation. The maximum increase in free intracellular calcium was observed with high concentrations of U46619. Notably, while low U46619 has limited effects on intracellular calcium, a sustained increase was observed adding epinephrine to platelet preparations (**Figure 2** and **Table 2**). The effects of 1 μM U46619 are partly dependent on extracellular calcium entry, as shown in the experiments with EGTA (**Figure 3**) (Sage and Rink, 1987). The contribution of calcium entry to the increase in free intracellular calcium was evident when 50 nM U46619 plus 10 μM epinephrine were tested. This finding is in agreement with previous observations indicating that epinephrine increased both the rate and amplitude of the rise in cytosolic free calcium in response to sub-threshold concentrations of thrombin and PAF, independently from the engagement of the fibrinogen receptor and platelet aggregation (Powling and Hardisty, 1988).

Consistently with the observation that Src phosphorylation is not required for thrombin-induced calcium entry (Harper and Sage, 2006), calcium increase is not altered by PP2 (**Table 4**) in our model (Dorsam et al., 2005; Harper and Sage, 2006).

To define which G proteins were implicated in free intracellular calcium increase downstream TP we compared the effects of different doses of U46619 with those of 8 iso-PGF_2α_, capable of inducing platelet shape change, but not platelet secretion and aggregation (Minuz et al., 1998). Previous investigation consistently demonstrated that platelet functional responses to 8-iso-PGF_2α_ implicate G_13_-coupled TPα receptor and P38 Mitogen Activated Protein Kinase (MAPK) (Minuz et al., 2002; Zhang et al., 2006). This was confirmed by the pharmacodynamic analysis of G-protein activation by thromboxane analogs, showing that 8 iso-PGF_2α_ displays high affinity for TPα, but is unable to activate Gq (EC_50_ 210 μM) while activating G_13_ (EC_50_ 34 μM). U46619 activates at similar concentrations both G_13_ and Gq (EC_50_ 31 μM and 39 μM, respectively) (Zhang et al., 2009). However, functional responses both to 8 iso-PGF_2α_ and U46619 are observed in the nanomolar range of concentrations, indicating that platelet activation occurs also in the presence of a receptor activation with limited downstream intracellular signals (Minuz et al., 1998, 2002; Zhang et al., 2009). The dose-dependency of calcium increase with U46619 is consistent with differences in Gq activation downstream TPα. Recently the role of focal adhesion kinase Pyk2 has been demonstrated to link calcium signals and Src activation (Canobbio et al., 2015), giving a plausible explanation also for the hierarchy in the signaling events we observed in U46619-stimulated platelets.

The addition of epinephrine, which does not *per se* increase intracellular calcium, potentiates the effects of low doses U46619. This is consistent with a model in which epinephrine synergises with U46619 to promote a sustained calcium response, through an altered channel activity by reducing the basal, cAMP-dependent phosphorylation of InsP3 receptors, increasing their responsiveness to agonists (Li et al., 2003; Hardy et al., 2004; Xiang et al., 2010).

**FIGURE 7:**
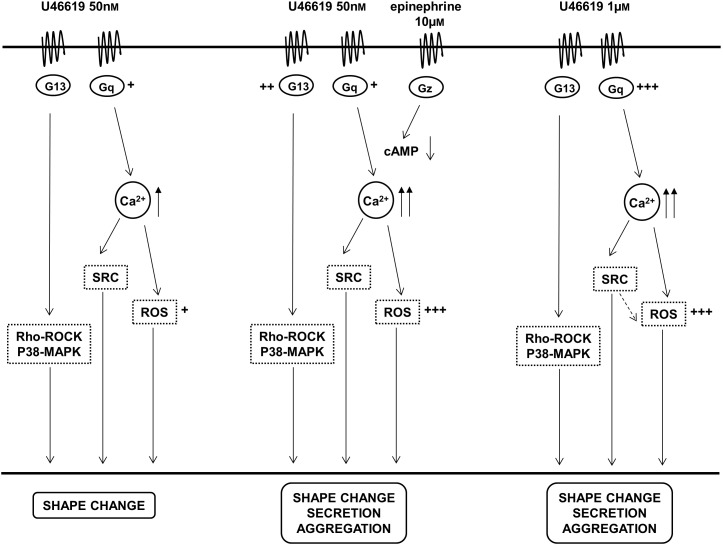
Graphs summarize the relationships between TP receptor activation, signaling pathways and functional responses in platelets stimulated with different amounts of the TXA_2_ analog U46619 and the cooperative effects of epinephrine. ^+^, ^++^, ^+++^ Represent differences in the implication of the signaling pathways.

We can define a threshold concentration of free intracellular calcium that is required to induce Src activation and a second level that is required for the release reaction and the α_IIb_β_3_ activation (**Figure 7**). This is demonstrated by the ability of PP2 or BAPTA to prevent all these functional events and by the evidence that 8-iso-PGF_1α_ does not synergize with epinephrine to increase intracellular calcium (**Table 4**). When high doses (1 μM) of U46619 are tested, most of the functional responses, except for granule secretion, in washed platelets are dependent on ADP released from dense granules (Minuz et al., 2006). Nevertheless, Src pathway is not inhibited, being crucially implicated in the residual secretion of the α granules and activation of the fibrinogen receptor that is observed in the presence of apyrase (Gratacap et al., 2009). This is also consistent with the observation that restoring ADP reverses the inhibitory effects of PP2 in thrombin and TXA_2_ stimulated platelets (Li et al., 2010b).

Calcium-dependent ROS generation represents a crucial step in platelet activation as a signal to elicit functional responses to U46619 in platelet. These are equally blunted by a scavenger of ROS and by the NOX inhibitor apocynin, thus demonstrating that these enzymes are responsible for ROS generation. Calcium is implicated in the release of ROS, since generation is reduced following store depletion (Rosado et al., 2001).

Seven NOX family members are so far been discovered in mammalian cells. Only two isoforms have been identified in platelets: NOX2 is the main catalytic unit present in platelets, but recently a role for the isoform NOX1 has been reported (Walsh et al., 2014). Platelet agonists like thrombin (Wachowicz et al., 2002), collagen (Pignatelli et al., 1998), and thromboxane analog U46619 (Begonja et al., 2005) induce ROS generation. ROS act as signaling mediators in platelets activation (Krötz et al., 2002). Our results indicate that NOX contributes to ROS production in stimulated platelet and is implicated in the activation of α_IIb_β_3_ integrin and the release reaction, independently of aggregation and integrin-dependent amplificatory signals. The NOX inhibitor has no effects on ROS generation in resting platelets, while calcium signals are required both for basal and stimulated ROS generation. However, only NOX5 has so far been shown to be calcium-dependent (Pandey et al., 2011). It has been demonstrated that the synthesis of ROS by oxLDL/CD36 in platelets requires Src-family kinases and protein kinase C (PKC)-dependent phosphorylation and activation of NOX2 (Magwenzi et al., 2015). More recently, it has been demonstrated that the NOX1 and the NOX2 knock-out mice exhibit distinct platelet response to agonists. In fact NOX1 -/y show specifically hyporesponsiveness to U46619 and thrombin, both acting through G protein-coupled receptors, resulting in blunted platelet aggregation and reduced ADP release (Delaney et al., 2016). Cumulating our observations with those obtained in the murine model, NOX1-derived ROS in platelet activation can be located downstream TP receptor, with amplificatory effects on calcium and tyrosine-kinase signaling response. Since apocynin does not inhibit ROS generation in resting platelets, we speculate that mitochondria are mostly involved in ROS generation in the absence of platelet stimulation. We also observed a distinct role of tyrosine kinases in collagen-dependent ROS generation, as demonstrated using dasatinib *in vitro*, confirming the contribution of signals downstream GPVI-ITAM in the activation of NAPDH oxidase activation (Qiao et al., 2018). This was not observed in G-protein coupled TP and adrenergic receptor depended ROS generation. A limitation in our study is the lack of in depth mechanistic investigation.

We conclude that TXA_2_ analogs that cause a partial occupation of the TP receptor and activation of a G13-dependent pathway, such as 8-iso-PGF_2α_, have limited effects on platelets inducing shape change and increased adhesion (Smyth, 1996; Minuz et al., 1998). When a Gq-dependent calcium signal is induced (even if limited, also in cooperation with a Gz-dependent pathway), platelet secretion and aggregation are induced, also through further activation of the Src kinase pathway. High doses of TXA_2_ activate a Gq pathway inducing platelet activation independently of the Rho kinase pathway, but this does not make redundant the signals ensuing from phosphorylated Src. In our opinion, the proposed model of platelet activation induced by TXA_2_ analogs effectively explains all the functional events deriving from TP occupancy. Our study mostly addresses the functional role of the main signaling pathways downstream TP using specific inhibitors to identify changes in platelet responses. The cooperative effects of epinephrine on platelet activation by low amounts of a Thromboxane analog suggests mostly explanations for biological phenomena that could be of clinical relevance rather than specific targets of pharmacological intervention. Further mechanistic and pharmacological investigation is advisable.

## Author Contributions

All authors contributed to the study and the preparation of the manuscript. PM and GB designed the study and wrote the manuscript. AM coordinated the laboratory investigation, analyzed data and performed the aggregation tests, the assays of intracellular calcium and ROS, as well as protein analysis along with LF and RF. MD performed the cytofluorimetric analyses. MR and DV collaborated to study design, patients selection, data analysis, and manuscript preparation.

## Conflict of Interest Statement

The authors declare that the research was conducted in the absence of any commercial or financial relationships that could be construed as a potential conflict of interest.
